# A rare case of ectopic partial molar pregnancy following IVF

**DOI:** 10.5935/1518-0557.20230030

**Published:** 2023

**Authors:** Sedighe Hosseini, Nazanin Hajizadeh, Elena Ghotbi, Mona Esmi

**Affiliations:** 1 Preventative Gynecology Research Center, Shahid Beheshti University of Medical Sciences, Tehran, Iran; 2 Division of Obstetrics and Gynecology, Alborz University of Medical Sciences, Karaj, Iran

**Keywords:** ectopic pregnancy, IVF, infertility, mole

## Abstract

A 29-year-old female who received assisted reproductive therapy (IVF) in our infertility clinic, at gestational age of 7w + 2d following embryo transfer, presented with a favorable rise of β-hCG level with no detectable gestational sac in the uterine cavity in the vaginal ultrasonogram. First dose of MTX (78) with simultaneous β-hCG titration of 110,000 pg/mL was administered. The patient underwent a second TVS in which a mass in favor of molar ectopic pregnancy was reported. With the suspicion of a molar EP the patient underwent explorative laparotomy. A 3x4 cm mass which was found adjacent to the right ovary was resected. Final pathology report was compatible with partial molar pregnancy. In the follow up period after surgical resection the patient recovered completely without any recurrence.

## INTRODUCTION

According to existing literature, apart from the rarity of ectopic molar pregnancy, its occurrence following assisted reproductive technology is exceedingly uncommon.

## CASE PRESENTATION

Here, we aim to report on a tubal partial molar pregnancy after In Vitro Fertilization (IVF). A 29-year-old female with a history of endometriosis and primary infertility was referred to our clinic seeking fertility treatment. Her partner had a diagnosis of abnormal sperm morphology and subsequent infertility. The patient had also a history of laparoscopy due to persistent ovarian cyst a year before IVF, which was pathologically reported as serous cyst adenoma. She had a case of well-controlled hypothyroidism; her past medical history was otherwise unremarkable, and her medications included only prenatal supplements and levothyroxine. She had decided to undergo IVF treatment and embryo transfer (ET).

Following ET serial β-hCG titers were monitored and at day 40 following ET (gestational age of 7w + 2d) she presented to our clinic with an appropriate rise of quantitative β-hCG (23,400 pg/mL). She reported to have no abdominal pain and vaginal bleeding. Her examination revealed a soft abdomen and her pelvic examination had no abnormal findings. A comprehensive TVS was performed and the findings included endometrial thickness of 7 mm and a 27x21 mm hyperheteroechoic lesion with peripheral vascularization adjacent to the right ovary in favor of an ectopic pregnancy.

According to elevated β-hCG level (110,000 pg/mL) and findings of ultrasound suggestive of EP, following consultation with the patient, methotrexate (MTX) therapy was initiated. A 78 mg of intramuscular MTX (50 mg/m^2^ of body surface area) was administered.

**Figure 1:**
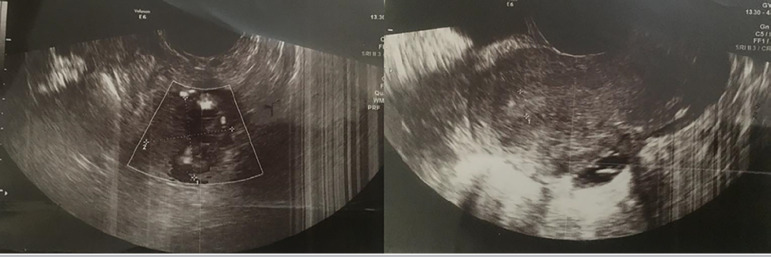
TVS revealing mass suggestive of molar ectopic pregnancy.

The patient received a second TVS by a fertility subspecialist in order to conduct a thorough investigation, in which a heteroechoic mass with central cysts and hyperheteroechoic border with multiple cysts in favor of partial molar EP was reported (Figure 1).

**Figure 2:**
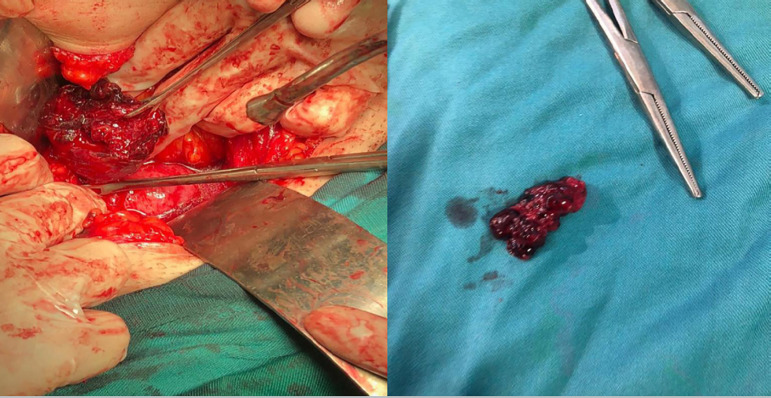
Intraoperative macroscopic view.

**Figure 3:**
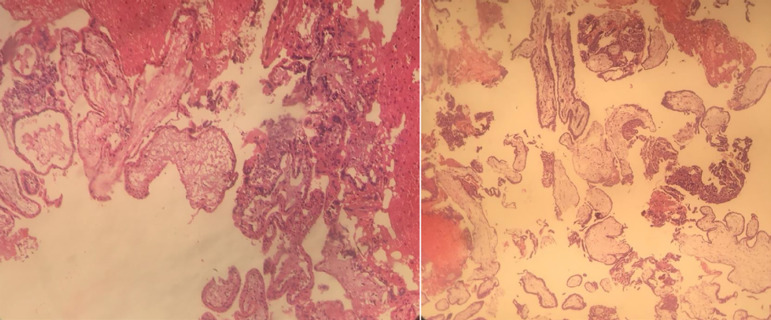
Microscopic findings suggestive of a partial mole.

With a suspicion of molar EP, the decision was made to proceed with explorative laparotomy and simultaneous dilatation and curettage. The intra-operative findings included uterus with extensive adhesion to the intestines and left tube and left ovary and a 3x4 cm mass adherent to the right ovary and right tube (Figure 2). The mass was excised and sent for a frozen section and was reported as probable molar EP. Final histologic report confirmed the diagnosis of a partial mole. (Figure 3)

Twenty-four hours following surgery, the β-hCG level fell to 11,000 pg/mL and at the7^th^ day post-surgery was reported to be 450 pg/mL. The patient continued weekly β-hCG level monitoring until three consecutive tests came back negative from three weeks post-op onwards. She was reported to have no recurrence of the disease over a follow-up period of 2 years.

## DISCUSSION

Ectopic molar pregnancy following Assisted Reproductive Therapy (ART) is an extremely rare entity. Hydatidiform moles arises due to abnormal fertilization of the ovum and sperm. It appears in two different forms: complete and incomplete (Allen *et al*., 2016; Bousfiha *et al*., 2012; Chauhan *et al*., 2004). In the incomplete mole, the chromosomal complement is diploid, with the genome being paternal in origin; while partial moles arise from a triploid genome (Hwang *et al*., 2010). Molar pregnancy has a predilection for the extremities of reproductive age range. A 15-year review from the Sheffield trophoblastic disease center showed that ectopic molar pregnancy affects 1.5 in every 1,000,000 pregnancies (Gillespie *et al*., 2004). It has been reported that the prevalence of ectopic pregnancy in the fallopian tube, ovaries, uterus horn, peritonea, cervix, and cesarean scar are 61%, 16%, 10%, 6%, 3% and 3%, respectively (López *et al*., 2018). Two major risk factors of ectopic pregnancy present in our case are endometriosis and ART related factors.

Several studies have shown that technical aspects of IVF are highly associated with increased risk of EP such as assisted hatching, frozen embryo transfer, higher transfer volume, deep fundal transfer and multiple embryo transfer (Obeidi *et al*., 2015). Furthermore, since the degree of trophoblastic proliferation is often more florid in EP, the histologic evaluation of ectopic molar pregnancy could be challenging. Also, there is no distinctive difference in β-hCG levels between ectopic mole and ectopic non-molar pregnancies (Hwang *et al*., 2010). In particular, an early ectopic molar pregnancy is not distinguishable from a non-trophoblastic EP (Obeidi *et al*., 2015). Occurrence of partial mole after IVF could be due to additional sperm being inserted into the ovum, also theoretically, triploidy could be the result of fertilization by a diploid sperm which is more common in infertile men as in our case (Hwang *et al*., 2010).

Histologically partial molar pregnancies are characterized by heterogeneity in villous size, enlarged irregularly shaped villi with scalloped borders and secondary trophoblastic pseudo-inclusions. As those cases are very rare, so reliance on only histology may lead to overdiagnosis. Also, discrimination between partial mole and complete mole can be difficult, so ploidy investigation could be helpful (Allen *et al*., 2016; Bousfiha *et al*., 2012; Chauhan *et al*., 2004; Obeidi *et al*., 2015). Patients with ectopic molar pregnancy more frequently present with symptoms of EP rather than intrauterine mole. But our case had no signs and symptoms of mole or EP. She was diagnosed in the follow up course by TVS and β-hCG level. In our case the clinical course of patient and sonographic, macroscopic and microscopic findings were suggestive of ectopic partial molar pregnancy, which was treated completely after surgery without any recurrence (Allen *et al*., 2016; Obeidi *et al*., 2015).
